# Induction of a Regulatory Phenotype in CD3+ CD4+ HLA-DR+ T Cells after Allogeneic Mixed Lymphocyte Culture; Indications of Both Contact-Dependent and -Independent Activation

**DOI:** 10.3390/ijms18071603

**Published:** 2017-07-24

**Authors:** Anne Louise Schacht Revenfeld, Rikke Bæk, Malene Møller Jørgensen, Kim Varming, Allan Stensballe

**Affiliations:** 1 Department of Clinical Immunology, Aalborg University Hospital, Urbansgade 32-36, DK-9000 Aalborg, Denmark; rikke.baek@rn.dk (R.B.); maljoe@rn.dk (M.M.J.); kv@rn.dk (K.V.); 2 Department of Health Science and Technology, Aalborg University, Laboratory for Medical Mass Spectrometry, Fredrik Bajersvej 7E, 9100 Aalborg, Denmark; as@hst.aau.dk

**Keywords:** peripheral tolerance, regulatory T cells, flow cytometry, EV Array

## Abstract

Although the observation of major histocompatibility complex II (MHCII) receptors on T cells is longstanding, the explanation for this occurrence remains enigmatic. Reports of an inducible, endogenous expression exist, as do studies demonstrating a protein acquisition from other cells by mechanisms including vesicle transfer. Irrespective of origin, the presence of the human MHCII isotype, human leukocyte antigen DR (HLA-DR), potentially identifies a regulatory T cell population. Using an allogeneic mixed lymphocyte culture (MLC) to induce an antigen-specific immune response, the role of antigen-presenting cells (APCs) for the presence of HLA-DR on cluster of differentiation 3(CD3)+ CD4+ T cells was evaluated. Moreover, a functional phenotype was established for these T cells. It was demonstrated that APCs were essential for HLA-DR on CD3+ CD4+ T cells. Additionally, a regulatory T cell phenotype was induced in CD3+ CD4+ HLA-DR+ responder T cells with an expression of CD25, CTLA-4, CD62L, PD-1, and TNFRII. This phenotype was induced both with and without physical T cell:APC contact, which could reveal novel indications about its functionality. To further investigate contact-independent communication, a phenotype of the small cell-derived vesicles from the MLCs was determined. Yet heterogeneous, this vesicle phenotype displayed contact-dependent differences, providing clues about their intended function in cellular communication.

## 1. Introduction

The expression of major histocompatibility complex II (MHCII) is essential for the specificity of the adaptive immune system. The T cell receptor (TCR) of CD4+ T cells interacts with the cognate peptide-MHCII complexes on antigen-presenting cells (APCs), thus activating the T cells. The constitutive expression of MHCII is limited to professional APCs, however, MHCII has for more than four decades also been observed on T cells [1,2,3,4]. In addition, the number of MHCII+ T cells increases upon activation [4,5]. In line with this, the most frequently expressed MHCII molecule in humans, the human leukocyte antigen DR (HLA-DR), is commonly used as a marker for T cell activation [5,6,7]. It remains a subject of discussion, whether the presence of MHCII on T cells is explained by an endogenous protein synthesis or by an acquisition from adjacent cells. Furthermore, the functional consequences of MHCII on T cells are still poorly understood.

An inducible, endogenous protein synthesis of MHCII has been suggested for T cells, similar to that described for APCs. The expression of MHCII has been observed for T cells from several species, including bovine [8], rat [9,10], and human [11]. In addition, T cells can also acquire fully functional MHCII from neighboring cells by yet unconfirmed mechanisms. This molecular transfer was already observed in the 1980’s [12] and since then several studies have demonstrated that the acquisition is possible for rat [13], mouse [14,15,16] and human T cells [17]. Moreover, it has been stated that an acquisition explains the presence of MHCII on mouse T cells [13,15], which are known not to express MHCII endogenously even after in vitro stimulation [18]. The responsible mechanisms have been reported as either being a vesicle-mediated transfer [13,19,20] or the acquisition of entire membrane patches [21], also known as trogocytosis [22]. Regardless of an endogenous expression or a protein acquisition, MHCII on T cells has mainly been associated with induction of down regulatory signals in the responding T cell [11,23,24,25]. It has also been associated with active rather than resting regulatory T cells (T_regs_) [26]. However, several studies also demonstrate that the MHCII+ T cells can activate other T cells [15,17]. Nonetheless, these functional findings collectively suggest a more pronounced role of MHCII on T cells than being an activation marker and more studies are required to investigate this.

The purpose of this study was to investigate if a functional and regulatory phenotype could be induced in human CD3+ CD4+ HLA-DR+ T cells by vesicles. An allogeneic mixed lymphocyte culture (MLC) was applied to induce an in vitro antigen-specific immune response. Since APCs have been shown to produce immunomodulatory vesicles [27,28,29] and play a role for HLA-DR on T cells, APCs were central in this investigation. As a new approach a contact-independent MLC was included, in which the allogeneic T cells and APCs were separated. This allowed for the study of several features of an antigen-specific immune response, which depend on the physical T cell:APC contact, including vesicle-mediated communication and the presence of HLA-DR on CD3+ CD4+ T cells. A functional phenotype was established for the HLA-DR-presenting T cells to clarify the conflicting observations about their function and to compare any effects of the T cell:APC contact on this. Vesicles have emerged as important immune regulators to induce functional phenotypes and also for intercellular transfer of proteins and other molecules [27,29]. Therefore, the phenotype of vesicles, here focusing on the small cell-derived extracellular vesicles (sEVs), was of interest, particularly when physical contact between T cells and APCs was absent. Consequently, an extensive phenotype was also determined for the cell-derived sEVs.

## 2. Results

### 2.1. Cellular Phenotype of HLA-DR-Presenting Responder CD3+ CD4+ T Cells

**Figure 1:**
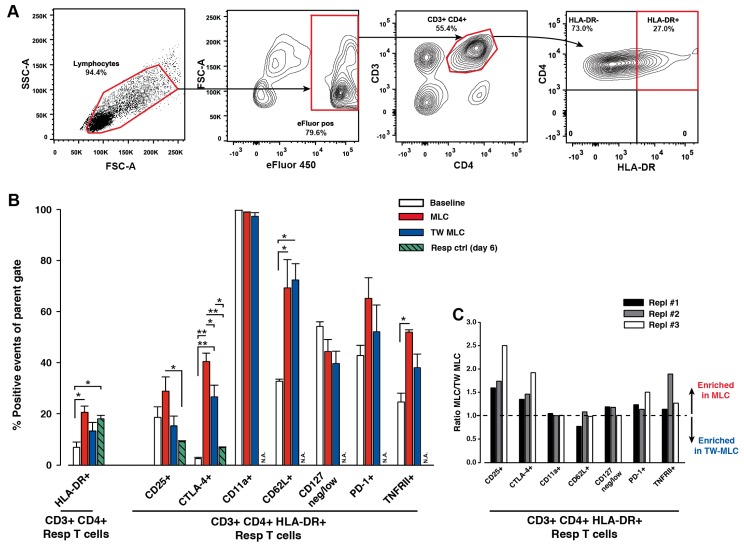
Cellular phenotypes of HLA-DR+ responder CD3+ CD4+ T cells after contact-dependent and -independent MLC. A flow cytometric analysis was used to determine the presence of HLA-DR and other selected cell surface markers on the responder cells of the contact-dependent MLC (classic) and contact-independent MLC (TW). (**A**) Gating of the responder T cells. The responder cells were identified from their eFluor 450 labeling, which separated them from the stimulator cells. This labeling was only used for separating responder cells from stimulator cells and not for proliferative measurements. HLA-DR+ events were identified with a pre-defined gate from a fluorescence minus one (FMO) control. The plots are representative examples from one of the three included biological replicates. (**B**) To compare their cellular phenotype, a flow cytometric evaluation of seven markers was performed at baseline (day 0) and at day 6 for the responder HLA-DR+ T cells from the classic MLC and the TW MLC. Selected markers were also investigated for the responder control at day 6. Data is presented as mean ± SEM. *n* = 3 (biological replicates; see Section 4.3). NA: Not available; this data was not determined. (**C**) A ratio of the expression of each of the seven markers was made between the classic MLC and the TW MLC. Repl: Replicate; Relates to each of the three biological replicates included. CTLA-4: cytotoxic T-lymphocyte associated protein 4. PD-1: Programmed cell death 1; TNFRII: TNF receptor II. *, *p* < 0.05; **, *p* ≤ 0.01.

A 6-day, one-way allogeneic MLC assay was employed to further investigate the importance of APCs for CD3+ CD4+ T cell activation and a subsequent presence of HLA-DR on CD4+ T cells. This assay induces an in vitro immune response with activation of the 5–10% responder CD4+ T cells that recognize alloantigens on the stimulator cells, which are irradiated and thus incapable of cellular activation and proliferation. The high frequency of alloreactive T cells, compared to T cells specific for a single antigen, enables a detectable response. Consequently, the assay is very useful for the in vitro study of various aspects of T cell activation and function [30]. The importance of a physical interaction between the responder cells and the stimulator cells was evaluated, using a contact-independent MLC (transwell (TW) MLC), in which a porous membrane physically separated the responder and stimulator cells, allowing only for transmembrane communication. The cellular phenotype of the CD3+ CD4+ HLA-DR+ responder cells was characterized by a flow cytometric evaluation of seven cell surface markers related to the activity and function of both T_regs_ and effector T cells (T_effs_). Initially, the CD3+ CD4+ HLA-DR+ T cells were identified (Figure 1A), using a fluorescence minus one (FMO)-approach [31]. As previously described, this enables a less user-biased placement of a positive gate, when a marker is expressed continuously, such as HLA-DR on CD3+ CD4+ T cells [32]. For the three culture samples, the levels of CD3+ CD4+ HLA-DR+ T cells were relatively comparable, although the fraction of these T cells was highest in the contact-dependent MLC (Figure 1B). Here, 20.5 ± 2.4% CD3+ CD4+ HLA-DR+ T cells could be detected, which was significantly different from the baseline measurements of 6.9 ± 2.0% (*p* = 0.009; *n* = 3, biological replicates). For the TW MLC and the responder control sample, 13.3 ± 3.3% and 18.0 ± 1.3% CD3+ CD4+ HLA-DR+ T cells were identified, respectively. The associated *p* values, when compared to baseline, were 0.174 and 0.022, respectively.

When comparing the phenotype of the HLA-DR-presenting CD3+ CD4+ responder T cells from the classic MLC and the TW MLC, four of the seven included markers were enriched in the classic MLC (Figure 1C). The markers included CD25, cytotoxic T-lymphocyte associated protein 4 (CTLA-4), tumor necrosis factor receptor II (TNFRII), and programmed cell death 1 (PD-1) (Figure 1B). The most differentially expressed marker was CD25, which exhibited almost a 2-fold increase in expression in the classic MLC, as compared to the TW MLC. In addition, the detected CD25 expression in the classic MLC was more than 3 times greater than the corresponding expression in the responder control (9.0 ± 0.4%; *p* = 0.027) and 1.6 times greater than the baseline expression (18.6 ± 4.1%). For the TW MLC, these numbers were 1.7 and 0.8, respectively. For CTLA-4, the expression was approximately 50% increased in the classic MLC, when compared to the observed expression in the TW MLC (40.5 ± 3.3% and 26.6 ± 4.6%; *p* = 0.017). The expression of CTLA-4 in both MLCs was also significantly different from the responder control (6.8 ± 0.3%), yielding a 4–6 times higher percentage-wise expression in the MLCs. Moreover, when compared to the baseline measurements (2.6 ± 0.4%), the expression was 10- and 15-fold higher in the TW MLC and classic MLC, respectively. With regard to TNFRII, the expression was approximately 40% greater in the classic MLC as compared to the TW MLC (51.9 ± 0.9% and 38.0 ± 5.4%) (Figure 1B). However, the CD3+ CD4+ HLA-DR+ responder T cells had increased the expression of TNFRII almost 1.6 and 2 times in the TW MLC and classic MLC, respectively, as compared to the baseline measurements (24.6 ± 3.5%). Finally, the expression of PD-1 was on average 25% greater in the classic MLC than in the in the TW MLC (65.2 ± 8.1% and 52.1 ± 10.4%). In relation to the baseline measurements (42.8 ± 4.0%), PD-1 expression was 1.2 and 1.5 times greater in the TW MLC and classic MLC, respectively. The observed differences for the percent-wise distribution of PD-1 was, however, not statistically significant between the three groups (Figure 1B; *p* = 0.145). As a final note, CD62L was highly expressed on the CD3+ CD4+ HLA-DR+ in both MLC (69.3 ± 11.2%) and TW MLC (72.4 ± 6.4%). When compared to baseline (32.7 ± 0.8%), the observed differences were statistically significant, with associated *p*-values of 0.025 and 0.019 for the MLC and TW MLC, respectively.

### 2.2. Differential Expression Separates the HLA-DR- and HLA-DR+ Responder CD3+ CD4+ T Cells

**Figure 2:**
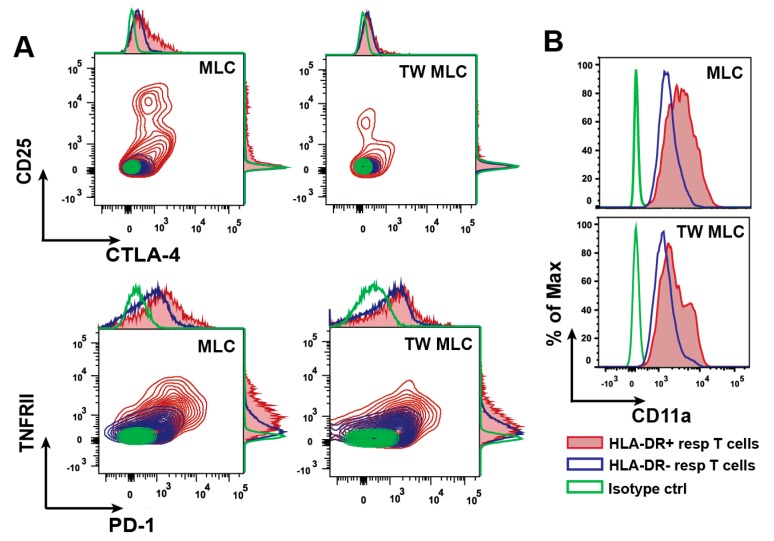
Differential expression of several surface markers on HLA-DR+ and HLA-DR- responder T cells determined by flow cytometry. (**A**) The correlation plots with adjunct histograms show the correlation between CTLA-4 and CD25 (top panel) as well as PD-1 and TNFRII (bottom panel) for CD3+ CD4+ HLA-DR+ responder T cells and the HLA-DR- equivalent. The plots are representative examples for the three biological replicates included; (**B**) The expression of CD11a shown for CD3+ CD4+ HLA-DR+ and CD3+ CD4+ HLA-DR- responder T cells. The histograms are representative for one of the three biological replicates included.

In order to further characterize the MLC responder cells, the phenotypical differences between the CD3+ CD4+ HLA-DR- and CD3+ CD4+ HLA-DR+ T cells were evaluated. As shown in the correlation plots in Figure 2A, four of the investigated markers were enriched in the HLA-DR+ subsets of both MLCs, as compared to the corresponding HLA-DR- subsets. These markers were CD25, CTLA-4, TNFRII, and PD-1. CD62L, which was highly expressed by the CD3+ CD4+ HLA-DR+ T cells from both MLCs (Figure 1B), was present in similar levels in the HLA-DR- subsets (MLC: 74.4 ± 15%; TW MLC: 70.2 ± 9). Since it was observed that PD-1 and TNFRII were present on both the HLA-DR+ and the HLA-DR- CD4 subsets (Figure 2A, bottom panel), an additional analysis was performed to describe the two T cell populations. Here, the median fluorescence intensity (MFI) values for the HLA-DR+ and HLA-DR- T cell populations were determined. For PD-1, the MFI values were on average 1.7 higher for the HLA-DR+ T cells (2400 ± 660), when compared to the HLA-DR- T cells (1400 ± 450) in the MLC. A similar observation could be found for the TW MLC, with a 1.4 times greater MFI value for the HLA-DR+ T cells (2100 ± 360) compared to the HLA-DR- T cells (1550 ± 370). Moreover, the observed differences in MFI were statistically significant (MLC *p* = 0.043; TW MLC, *p* = 0.009; *n* = 3, biological replicates). A comparable trend could also be detected for TNFRII. In the MLC setting, the associated MFI values for the CD3+ CD4+ HLA-DR+ T cells (1000 ± 110) were on average 2.2 times greater than the MFI values for the corresponding CD3+ CD4+ HLA-DR- T cells (450 ± 30). This number was 1.6 for the TW MLC, when comparing the MFI values for HLA-DR+ T cells (740 ± 25) with those for the HLA-DR- T cells (475 ± 30). Also here, the observed MFI differences were statistically significant (MLC *p* = 0.024; TW MLC, *p* = 0.004). As a final notion, it was consistently observed for both types of MLC that the expression of CD11a was upregulated on the CD3+ CD4+ HLA-DR+ responder T cells, as compared to the corresponding HLA-DR- subsets (*n* = 3) (Figure 2B).

### 2.3. Requirements for MLC-Induced Cellular Proliferation

**Figure 3:**
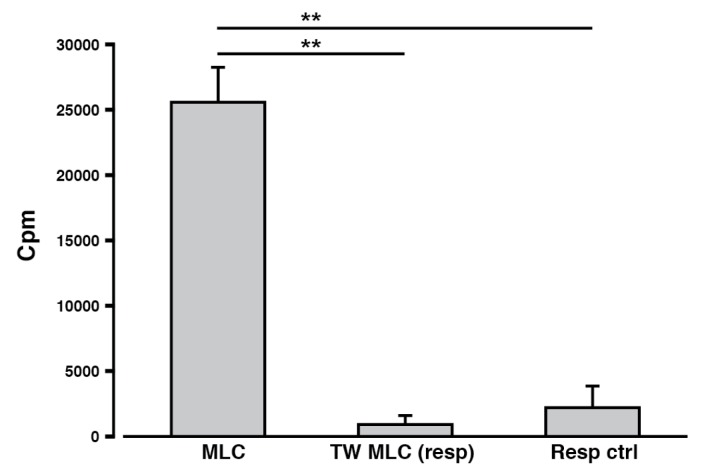
Cellular proliferation after contact-dependent and -independent MLCs. After a 6-day MLC, either contact-dependent (classic) or contact-independent (TW), the proliferation of the responder cells was determined. Data is presented as mean ± SEM. **, *p* ≤ 0.01; *n* = 3 (biological replicates). Cpm: Counts per minute.

As an additional measure, the cellular proliferation was determined for the two MLC combinations. This is a conventional outcome measure, as proliferation indicates cellular activation as a result of antigen recognition. A prominent proliferative response was detected from the cells in the contact-dependent MLC (classic MLC), as compared to the responder control sample (Figure 3). Contrary to this observation, the responder cells from the contact-independent MLC (TW MLC) did not proliferate above the level detected for the responder control. The contrasting proliferative profiles consequently either suggests that an allogeneic immune response was only initiated in the classic MLC or that differential cellular mechanisms and functionalities are associated with the two types of MLCs.

### 2.4. Inducible Presence of HLA-DR in Isolated CD3+ CD4+ T Cells

**Figure 4:**
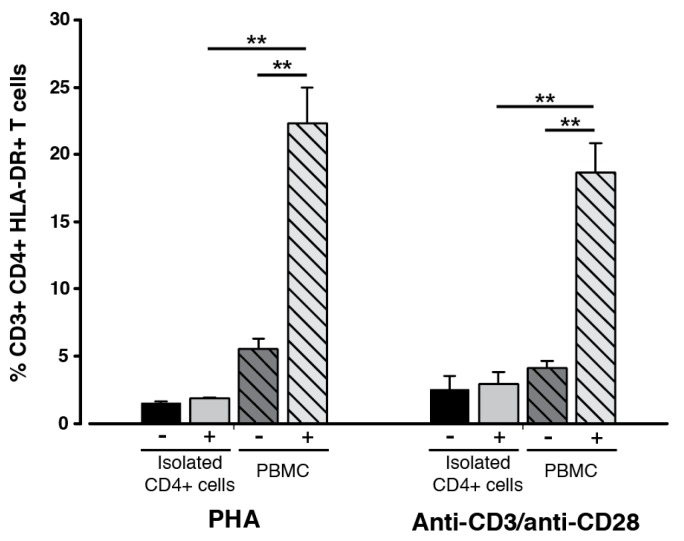
Presence of HLA-DR on in vitro phytohaemagglutinin (PHA) or anti-CD3/anti-CD28 stimulated CD3+ CD4+ T cells. Isolated CD4+ T cells or peripheral blood mononuclear cells (PBMCs) were stimulated with PHA or anti-CD3/anti-CD28 for 20 h. Subsequently, the presence of HLA-DR was evaluated by flow cytometry for the CD3+ CD4+ T cells in all samples. Data is presented as mean ± SEM. **, *p* ≤ 0.01; *n* = 2–6.

To further investigate the role of APCs for the presence of HLA-DR on CD3+ CD4+ T cells, we prepared monocultures of CD3+ CD4+ T cells from peripheral blood, thus removing the APCs. The T cell monocultures were then activated with either phytohaemagglutinin (PHA) or anti-CD3/anti-CD28 to induce a possible endogenous expression of the HLA-DR observed on their cell surface. These two types of stimuli activate T cells through different mechanisms and since HLA-DR has been termed a T cell activation marker it was expected that an increase in the surface-associated HLA-DR could be observed upon stimulation. However, for both types of stimuli, an increase in the presence of HLA-DR could not be detected after 20 h of stimulation for the isolated T cells (Figure 4, filled bars). In contrast, the number of CD3+ CD4+ HLA-DR+ T cells increased, when peripheral blood mononuclear cells (PBMCs) were stimulated with either PHA (*p* = 0.008) or anti-CD3/anti-CD28 (*p* = 0.001), as compared to the unstimulated counterpart (Figure 4, hatched bars). Moreover, the proportion of CD3+ CD4+ HLA-DR+ T cells was approximately 12 and 9 times greater in the PBMC population than in the monocultures stimulated with PHA (*p* = 0.006) or anti-CD3/anti-CD28 (*p* = 0.002), respectively. The purity of the isolated CD3+ CD4+ T cells was 94.5 ± 5.2% (*n* = 3), as evaluated by flow cytometry. Collectively, the observations suggest a non-existing HLA-DR expression in CD3+ CD4+ T cells and further underline a significant role of APCs for the presence of HLA-DR on the surface of CD4+ T cells.

### 2.5. Contact-Dependent Differences Observed for the Phenotype of Small Extracellular Vesicles Following Allogeneic MLC

As demonstrated above, APCs were essential for the presence of HLA-DR on CD3+ CD4+ T cells. Moreover, a regulatory phenotype could be induced in HLA-DR-presenting CD3+ CD4+ T cells (Figure 1), when physical T cell:APC contact was absent, pointing to a role of vesicles in this aspect. Therefore, we wanted to examine the cell-derived vesicles from the MLC cultures. This was achieved by a phenotypical characterization of the sEVs from the cell supernatants on day 6 of the MLCs. For the characterization, we applied the extracellular vesicle (EV) Array to detect the sEVs displaying CD9, CD63, and/or CD81; three vesicle-specific markers [33]. The EV Array is a semi-quantitative assay, which has been shown to analyze sEVs that are mainly <150 nm [34]. No additional quantification of the sEVs was performed, since the cells/volume in the cell culture setups were similar, enabling direct comparison between the levels of each marker detected in the MLC and TW MLC.

**Figure 5:**
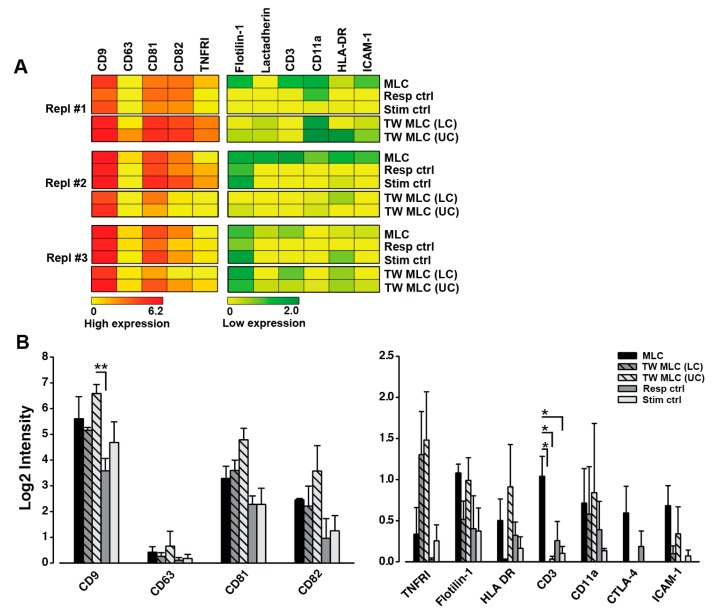
The phenotype of small extracellular vesicles (sEVs) from the MLCs. The extracellular vesicle (EV) Array was applied to extensively phenotype the sEVs from the cell supernatants of the MLCs. For the TW MLC, the upper chamber (UC) and lower chamber (LC) contained the stimulator cells and responder cells, respectively. Antibodies targeting the listed markers were used for capturing of the sEVs. The signal observed for each of the markers infers a simultaneous presence of CD9, CD63, and/or CD81, since a cocktail of antibodies against these three vesicle markers was used for detection. (**A**) Summary of selected, investigated markers for sEVs shown for each biological replicate; (**B**) The relative distribution of selected EV markers for all MLCs and controls are visualized for the highly expressed markers (left plot) and for those with a lower expression (right plot). Data is presented as the mean ± SEM of the three included biological replicates. *, *p* < 0.05; **, *p* ≤ 0.01.

A summary of the protein markers detected on these sEVs is depicted in the heat maps in Figure 5A. Of the three EV-specific markers, only CD9 and CD81 could be detected in all samples. In addition, CD82+ sEVs was also present in all samples. The relative distribution of these markers can be seen in the left bar plot in Figure 5B. Here, it can be noted that the difference in the level of sEV-associated CD9 detected in the upper chamber (UC) of the TW MLC samples, containing the stimulator cells, was statistically significant, when compared to the responder control (*p* = 0.006). The presence of the remaining markers included in the EV phenotyping was quite heterogeneous. However, tendencies were present for a number of these markers. As such, CD3 was predominantly detected in the sEVs from the classic MLC, while it was practically absent in the TW MLC samples (Figure 5B, right bar plot). There was a statistically significant difference between the sEV level of CD3 detected in the classic MLC as compared to the sEV CD3 detected in TW MLC lower chamber (LC), the TW MLC UC, and the stimulator control samples (*p* = 0.018, *p* = 0.021, *p* = 0.031, respectively) (Figure 5B, right panel). As for CD3, CTLA-4 was also primarily detected on the sEVs from the classic MLC. Nevertheless, the detectable signals for this marker were low. In the TW samples, sEVs were more enriched in TNFRI, when compared to the classic MLC and the controls (Figure 5B). In relation to HLA-DR, the obtained signals were all relatively low. However, this protein was mainly detected on sEVs from the classic MLC and the upper chamber of the TW MLC system, which contained the stimulator cells. Apart from the heterogeneous presence of many of the included markers, several of these markers could not be detected or yielded barely detectable signals for sEVs from the MLCs. These included lineage specific markers CD4 (T cells), CD19 (B cells), and CD83 (DCs) (data not shown). Moreover, the hematopoietic marker CD45 and the class I equivalent of HLA-DR, HLA-ABC, were also included in this list (data not shown). The applied growth medium was also included in the sEV analysis and yielded no detectable signal for any of the included markers (data not shown). Since the characterized sEVs relate to all the cells in the culturing system it was not possible to isolate any cell-specific information. However, it is a new discovery that the phenotype of sEVs produced during in vitro antigen-specific immune responses is highly heterogeneous.

## 3. Discussion

For many years it has been believed that T cells from most species possess the ability to express HLA-DR/MHCII endogenously, particularly following activation. However, this dogma has been challenged by several studies, demonstrating that a protein acquisition either completely account for or contribute to the presence of HLA-DR on T cells. Nonetheless, HLA-DR in the context of T cells points to a regulatory T cell subset, yet their potential functionality remains unclear. This formed the basis to investigate the induced functional phenotype of CD3+ CD4+ HLA-DR+ after an in vitro antigen-specific immune response. Here, the role of the physical contact with APCs and the cell-derived vesicles was examined.

Initially, the significance of the physical T cell:APC contact was addressed. For that, both a contact-dependent (classic) allogeneic MLC and a contact-independent MLC (Transwell, TW MLC) were performed. By employing a MLC the in vivo antigen-recognition by T cells was approximated [35] and the consequent and expected activation of CD3+ CD4+ responder T cells would enable an analysis of HLA-DR presentation by these cells. Moreover, establishing a functional phenotype for these cells would provide information about their potential role.

As can be visualized from Figure 1B, the fraction of the CD3+ CD4+ HLA-DR+ T cells were quite similar for the MLCs and the responder control, although an increase was observed from the baseline measurements. Hence, any suggestions about the mechanisms explaining the presence of HLA-DR on the CD3+ CD4+ could not be immediately explained with these observations. However, it is an important observation that the phenotype of these specific T cells from the MLCs was not induced as an artifact by the culturing conditions, as the MLCs created notably different CD25 and CTLA-4 expression compared to the control (Figure 1B). It has also been demonstrated that a certain degree of non-specific autoproliferation of CD4+ responder T cells, including T_regs_, can be expected during MLCs [36]. This may explain the percentage-wise increase in CD3+ CD4+ HLA-DR+ T cells in the responder control, when compared to baseline. All in all, this signifies that an actual cellular response was induced in both MLC scenarios. To further support this observation, we demonstrated that the presence of HLA-DR on CD3+ CD4+ T cells could not be induced by activation stimuli, when APCs were absent (Figure 4). Taken together, these results indicate an essential role of APCs, and possibly accessory cells, for HLA-DR on CD3+ CD4+ T cells.

Since a cellular response was seemingly induced in both the contact-dependent and –independent MLC, we addressed yet another aspect relating to the physical T cell:APC contact and T cell activation. A classic experimental readout from a MLC is to measure the cellular proliferation. A proliferative response during a MLC points to the induction of an antigen-specific immune response, in which T cells are activated and can differentiate into T_effs_ [15,30,37]. In this study, contact-dependent differences in the proliferative capacity could be observed, as only a notable proliferative response was detected for the responder cells in all contact-dependent (classic) MLCs (Figure 3). Hence, the proliferation observed in allogeneic MLCs required physical contact between T cell and APC. Since a cellular reaction was initiated in both MLCs, the lack of proliferation in the TW MLC could indicate the presence of an alternate cellular reaction compared to the classic MLC. This points to mechanistic, and most likely also functional, differences for the two MLC scenarios.

To search for these potential functional differences linked to the physical T cell:APC contact and to expand on the role of HLA-DR on CD3+ CD4+ T cells, a functional phenotype for the responder CD3+ CD4+ HLA-DR+ T cells was characterized. This characterization included seven cell surface markers, including general T cell activation markers, as well as markers associated with T_regs_ and T cell suppression. The latter category of cell surface markers was included since HLA-DR on T cells mainly has been associated with suppressive activity [23,24,25], as it has been observed for HLA-DR+ T_regs_ [11]. In addition, anti-MHCII antibodies have been demonstrated to block the suppressive activity of activated human T_regs_ [38], suggesting an important role for HLA-DR on T_regs_. Of the seven included markers, the differential expression was mostly pronounced for four of these, when comparing the classic MLC to the TW MLC. These included CD25, CTLA-4, PD-1, and TNFRII (Figure 1B) and all markers were mostly enriched in CD3+ CD4+ HLA-DR+ responder T cells from the classic MLC (Figure 1C). Moreover, the expression of the four selected markers was enhanced in the CD3+ CD4+ HLA-DR+ T cells, when compared to the corresponding HLA-DR- subsets (Figure 2A). Therefore, these markers could help to define the function of the HLA-DR+ T cells. Finally, a pronounced and comparable expression of one marker, CD62L, could be detected for the two MLCs. It is notable that the phenotype of the CD3+ CD4+ HLA-DR+ responder T cells in the TW MLC was almost identical to the corresponding phenotype in the classic MLC, although with a delayed or a less prominent expression (except for CD62L). This was demonstrated for all of the three included biological replicates, pointing to a consistent trend. The novelty in these observations is that a similar phenotype could be induced in the CD3+ CD4+ HLA-DR+ T cells both by direct contact with APCs (MLC) and by vesicles, which were likely produced by the APCs. It would consequently be interesting to explore if the TW MLC response is a component of the full T cell response observed in the contact-dependent, classic MLC or if it could represent a separate mechanism for immune regulation.

Regarding the significance of the cellular phenotyping, most of the investigated markers can be found on both conventional T_effs_ and T_regs_. Due to their dissimilar functionalities, it was important to look for expressional patterns to differentiate these two cellular groups. In the case of CD25 and CTLA-4, these two markers displayed a positive correlation for a part of the CD3+ CD4+ HLA-DR+ cells (Figure 2A, top panel). However, the non-uniform correlation demonstrated how the CD3+ CD4+ HLA-DR+ T cells were a heterogeneous cell population, which we have also previously observed [32]. Like CTLA-4, PD-1 has an essential role in T cell inhibition and consequently also in peripheral tolerance [39,40]. Moreover, T cells can express one of the two ligands for PD-1 (PD-Ls) [39,41]. A PD-1/PD-L1 ligation in T cells is mostly associated with induction of anergy [39,41,42,43]. Interestingly, the ligation between PD-1 and PD-L has also been suggested to play a role in the interaction between T cells [44], though with currently unverified effects. Blocking CTLA-4 and PD-1 reduces T_reg_ activity in melanoma patients [45] and, as previously mentioned, blocking of HLA-DR on activated T_regs_ has similar effects [38]. This could suggest a yet unknown link between HLA-DR, CTLA-4, PD-1, T_regs_, and their suppressive capacity. TNFRII was also enriched in the CD3+ CD4+ HLA-DR+ responder T cells in both MLCs, when compared to the HLA-DR- counterparts (Figure 2A, bottom panel). Moreover, TNFRII positively correlated with the expression of PD-1. Several studies have shown that TNFRII is primarily confined to T_regs_ in both human and mouse [46,47,48]. A suppressive activity of CD4+ CD25+ TNFRII+ HLA-DR+ T cells has been established with an observed co-expression of CTLA-4 [25]. The T cell homing receptor CD62L, or L-selectin, has been detected on CD4+ CD25+ HLA-DR+ T_regs_ in peripheral blood [11] and a suppressive capacity was demonstrated for CD4+ CD25+ CD62L+ T_regs_ [49,50]. Hence, a concurrent presence of HLA-DR, CD25, CD62L, CTLA-4, PD-1, and TNFRII on CD4+ T cells could very likely identify a subset of cells with suppressive activity. An identification of such a potential suppressive function of the CD3+ CD4+ HLA-DR+ responder T cells in the present study would consequently be relevant. In total, the observations presented here strongly suggest a role as T_regs_ rather than T_effs_ for the CD3+ CD4+ HLA-DR+ T cells.

As established, the physical T cell:APC contact influenced both proliferation and cellular phenotype. Hence, cellular communication by soluble factors and vesicles were central in the TW MLC setup and possibly also in the classic MLC. Previous reports demonstrate transfer of HLA-DR to T cells from APCs by vesicles [13,19,20]. In addition, EVs have emerged as important regulators in the immune system [29]. Based on this, we characterized the sEVs in the cell supernatants from the MLCs to obtain information about the vesicular communication and functionality. This was achieved by extensively phenotyping the sEVs for both vesicle-specific and cell-specific protein surface markers, using the EV Array [33,34]. Only vesicle surface membrane-associated proteins, and not soluble proteins, can be detected with this technology, due to the use of different antibodies for capture and detection. Moreover, crude cell supernatant can be applied without additional vesicle isolation, as constituents and vesicles inherently present in the growth medium alone do not yield any detectable signal [34,51,52].

With this analysis, we detected sEVs containing CD9, CD81, and CD82, from all MLCs and controls. Of these three tetraspanins, CD9 and CD81 are generally accepted as vesicle hallmark markers that identify a particular subset of sEVs, called exosomes [53,54]. However, these proteins are not exclusively present on this vesicle type. Moreover, CD82 has also been associated with this type of EV [55]. EVs, and in particular exosomes, are known to be key players in cellular communication, both in maintaining homeostasis and for the progression of pathological condition, including cancer [33]. Also, immunomodulatory effects of EVs have been documented, involving transfer of proteins and other molecules, such as RNA, between cells, effectively changing the phenotype and function of the recipient cell [19,29,56,57]. The detection of CD9, CD81, and CD82 in the current study indicated that production of such sEVs were present in all of the cellular samples. This is relevant, since vesicle-based cellular communication in the context of allogeneic MLCs has yet to be characterized. However, the detected sEV phenotypes were very heterogeneous across the biological replicates (Figure 5B, right bar plot). Although the phenotype was heterogeneous, this study is, to the best of our knowledge, the first to present sEV phenotype analyses from MLCs. It has been demonstrated that the sEV phenotype obtained in the applied TW setup is reproducible [51], thus pointing to other factors giving rise to this heterogeneity. Pronounced inter-individual variations in the sEV phenotype have been observed for the vesicles found in plasma of healthy individuals [34]. The quite different sEV phenotypes detected in the present study could be a consequence of such variations. This may indicate that the EV phenotype can be individually adapted, which is unlike the cellular phenotype described above, for which there were consistent trends for all individuals.

The heterogeneity of the sEV phenotypes complicates identification of their functionality. Nevertheless, a number of contact-dependent differences could be detected. Two T cell-associated proteins, CD3 and CTLA-4, were predominantly observed in the classic MLC. Conversely, TNFRI was mostly found on sEVs from the TW system. Also, HLA-DR-bearing sEVs were mostly observed in the TW compartment holding the stimulator cells. In terms of functionality, the down-regulation of CD3 following T cell activation [58,59] has been associated with the production of CD3-enriched EVs [60], as a tool of this down-regulation. Hence, EVs may be used for other purposes than intercellular communication. Though the contact-dependent differences in the sEV phenotypes were subtle, they could reflect distinct functions of these vesicles in each of the MLC systems. Nonetheless, this remains to be determined. In the context of HLA-DR, it has been noted that vesicles possibly mediate the demonstrated transfer of HLA-DR from APCs to T cells [15,16,17,19,20] and that they can perform direct antigen presentation to T cells [27,56,61,62]. The levels of sEV-associated HLA-DR detected for both MLCs were low. This may either signify a modest production of HLA-DR bearing sEVs or a high uptake rate, making them inaccessible to analysis. In spite of this, HLA-DR bearing vesicles were in fact produced, which could indicate a role for them in antigen-specific immune responses and also for HLA-DR on T cells. However, to fully support this, any by-stander effects from autologous APCs in the system should be eliminated and co-cultures of T cells and allogeneic APCs could be prepared. In addition, more studies are necessary to track whether such HLA-DR+ sEVs can be incorporated into T cells, either in a random or non-random fashion, and to investigate the potential functionality. Such studies could also assist to fully identify the source of the sEVs. Nonetheless, the sEV-related results point to a relevance of vesicle analysis in relation to in vitro induced antigen-specific immune responses, which may in turn provide novel insight into the in vivo immunomodulatory significance of EVs.

## 4. Materials and Methods

### 4.1. Cells and Isolation

Venous peripheral blood was obtained from healthy donors with known HLA types. Each blood donor had signed a written consent form, allowing for the use of his or her blood for research purposes. The procedure was approved by local ethics legislation. The blood was collected in heparinized tubes (Vacuette, Lithium Heparin, Greiner Bio One, Frickenhausen, Germany). Isolation of peripheral blood mononuclear cells (PBMCs) was accomplished by using Lymphoprep ™ gradient centrifugation (Axis-Shield, Oslo, Norway). The PBMCs were either used directly after the isolation or stored at −140 °C in a storage medium (RPMI 1640 (Gibco, Life Technologies, Carlsbad, CA, USA), 40% heat-inactivated fetal calf serum (FCS) (Gibco), 10% dimethyl sulfoxide (Merck Millipore, Darmstadt, Germany), 100 U/mL penicillin/10 µg/mL streptomycin (Ampliqon, Odense, Denmark)). The CD4+ T cells were isolated from PBMCs using either a Dynabeads^®^ CD4 Positive Selection kit (Invitrogen, Life Technologies, Carlsbad, CA, USA) or a Dynabeads^®^ Regulatory CD4+ CD25+ T Cell Kit (Invitrogen), using only the CD4+ isolation part. The entire procedure was carried out according to the manufacturer’s guidelines. The purity of the isolated cells was evaluated by flow cytometry.

### 4.2. Mitogenic and Antigen-Like Stimulation

The isolated CD4+ T cells or PBMCs were seeded in a concentration of 7 × 10^5^ cells/mL in culture medium (RPMI 1640, 10% FCS, 100 U/mL penicillin/10 µg/mL streptomycin) with or without mitogenic/antigen-like stimuli for 20 h. The mitogen PHA (Sigma-Aldrich, St. Louis, MO, USA) was used in a final concentration of 3.3 µg/mL, while Dynabeads^®^ Human T-Activator CD3/CD28 for T Cell Expansion and Activation (Invitrogen) was used according to the manufacturer’s guidelines.

### 4.3. Mixed Lymphocyte Culture

A 6-day, one-way allogeneic mixed lymphocyte culture (MLC) was performed with PBMCs from two donors with HLA mismatch, in order to induce an allogen-specific immune response. Each of the three included biological replicates comprised two unique donors, thus including six donors in total. Prior to the MLC, the stimulator cells were irradiated (1700 rad), while the responder cells were labeled with 15 μM Cell Proliferation Dye eFluor^®^ 450 (eBioscience, San Diego, CA, USA) according to the manufacturers guidelines. This labeling was performed to allow for separation of responder cells and stimulator cells in the flow cytometric analysis and was not used to evaluate the proliferative response (see below for specifics on proliferation). For the contact-dependent MLC (classical), 5 × 10^4^ of each responder and stimulator cells were mixed in a 96-well plate (Nunc, Thermo Scientific, Waltham, MA, USA) in a total volume of 150 µL culture medium (RPMI 1640, 10% FCS, 100 U/mL penicillin/10 µg/mL streptomycin). Approximately 96 wells were made for each classic MLC. For the contact-independent setup, stimulator cells and responder cells were separated by a Millicell^®^ Hanging Cell Culture Insert (Merck Millipore) with a pore size of 0.4 µm (hereafter termed transwell (TW)) in 24-well plates (Nunc). Since the relative placement of the cells in the TW system can affect the vesicle phenotype, the cells exerting the major stimulatory function (the stimulator cells) were placed in the UC of the TW system, while primary vesicle recipient (responder cells) was placed in the LC [51]. The upper chamber (UC) contained 2.5 × 10^5^ stimulator cells in 400 µL of culture medium, while the lower chamber (LC) contained 5 × 10^5^ responder cells in 800 uL of culture medium. A total of 10 wells were made for each TW MLC. For the stimulator and responder control samples, 5 × 10^4^ of either responder or stimulator cells were seeded in a 96-well plate, as described above. A total of approximately 24 wells were made for the control samples. On day 5, 50 µL (1 µCi, 96-well plate) or 400 µL (8 µCi, 24-well plate) of Thymidine-^3^H (Perkin Elmer, Waltham, MA, USA) was added to the selected wells designated for measurements of proliferation. The Thymidine-^3^H labeled cells were harvested after 24 h and radioactive incorporation was detected in a scintillation counter (TopCount NXT, Perkin Elmer, Waltham, MA, USA). On day 6 of the MLCs, the cell culture supernatants were removed and centrifuged once at 500× *g* 10 min at room temperature (RT) to pellet cells. A protease inhibitor cocktail (EDTA-free, diluted 1:50 in PBS) was added to the cell-free supernatants, which were stored at −40 °C prior to vesicle phenotyping. No further isolation of the EVs was performed. A cellular phenotyping of the cells from the MLCs was performed using flow cytometry.

### 4.4. Cellular Phenotyping with Flow Cytometry

Antibodies for cellular phenotyping: Conjugated antibodies against the following targets were obtained from BD Biosciences (Mountainview, CA, USA): CD3-APC (UCHT1), CD3-FITC (UCHT1), CD3-PerCP (SP34-2), CD4-APC-H7 (SK3), CD4-FITC (RPA-T4), CD4-PE (RPA-T4), CD11a-FITC (G43-25B), CD25-FITC (M-A251), CD62L-FITC (DREG-56), CD127-PE (HIL-7R-M21), CD152 (CTLA-4)-PE (BNI3), CD279 (PD-1)-PE-Cy7 (EH12.1), HLA-DR-FITC (L243), HLA-DR-PE (L243), HLA-DR-PerCP-Cy5.5 (L243) (these anti-HLA-DR antibodies were applied for the analyses shown in Figure 4 and only % Positive events, and not median fluorescence intensity (MFI) values, were used in the data analysis), mouse-IgG1-APC-H7 (X40), mouse-IgG1-FITC (MOPC-21), mouse IgG1-PE (MOPC-21), mouse IgG1-PerCP (MOPC-21), and mouse IgG1-PE-Cy7 (MOPC-21). Antibodies against HLA-DR-Alexa Fluor 647 (L243) (this anti-body was used for the analyses shown in Figure 1) and mouse-IgG2a-Alexa Fluor 647 (MOPC-173) were from BioLegend (San Diego, CA, USA). Mouse-IgG2a-FITC (eBM2a) and mouse-IgG2a-PE (eBM2a) were from eBioscience. Anti-TNFRII (CD120b)-PE (80M2) was from Immunotech (Beckman Coulter, Inc., Brea, CA, USA). Mouse-IgG1-APC (DAK-GO1) was from Dako A/S (Glostrup, Denmark).

Staining of cells: For the detection of selected surface markers, 1 × 10^6^ of harvested PBMCs from the MLCs were stained with the relevant antibodies or the matched isotype control antibodies (30 min, RT). After staining, the cells were washed once with PBS prior to the flow cytometric analysis.

Data acquisition and analysis: The acquisition of stained cells was performed on a FACSCanto A using FACSDiva™ software (version 6.1.3, BD Biosciences, Mountainview, CA, USA). Calibration and compensation settings for the cytometer were obtained daily by using the 7-Color Setup Beads (BD Biosciences, Mountainview, CA, USA) and once a week with the FACSDiva™ CS&T Research Beads (BD Biosciences, Mountainview, CA, USA). The analysis of the data was carried out with the FlowJo software (version 10.0.7, FlowJo LLC, Ashland, OR, USA). Negative isotype controls or fluorescence minus one (FMO) controls [31,63] were utilized to identify the positive events. For the FMO control a maximum of 1% of positive events were allowed in the double-positive quadrant.

### 4.5. EV Array Analysis

Microarray production: Epoxy-coated slides (Schott Nexterion, Jena, Germany) were printed using SpotBot^®^ Extreme Protein Edition Microarray Printer (ArrayIt, CA, USA) as previously described [52,64].

Antibodies and proteins for vesicle phenotyping: A total of 24 anti-human antibodies and one protein were used. They are listed in the following with the corresponding product number (#) or clone. From R&D Systems (Minneapolis, MN, USA): Annexin V (#AF399), CD4 (34930), CD19 (4G7-2E3), CD45 (2D1), CD80 (37711), CD82 (#423524), CD83 (H15e), LAMP-2 (H4A3), TNFRI (#DY225), and TNFRII (#DY726). From Biolegend: Alix (3A9), CD63 (MEM-259), HLA-ABC (W6/32), and HLA-DR (L243). From LifeSpan BioSciences, Inc. (Seattle, WA, USA): CD9 (#LS-C35418), CD81 (#LS-B7347), and CTLA-4 (ANC152.2/8H5). From BD Biosciences: CD3 (Hit3a) and CD14 (M5E2). From Abcam (Cambridge, MA, USA): Flotilin-1 (#Ab41927) and TSG101 (5B7). From Santa Cruz Biotechnologies (Dallas, TX, USA): TLR-3 (TLR3.7). From Abbiotec (San Diego, CA, USA): CD11a (HI111). From eBioscience: ICAM-1 (R6.5). From Haematologic Technologies, Inc. (Essex Juncton, VT, USA): Lactadherin (protein) (#BLAC-1200). All antibodies and protein were printed in triplicates at 180–200 µg/mL diluted in PBS containing 5% glycerol.

Catching and visualization: Blocking of the microarray slides was performed in 50 mM ethanolamine, 100 mM Tris, 0.1% SDS, pH 9.0 prior to incubation with 100 µL of undiluted cell culture supernatant. The incubation was performed in Multi-Well Hybridization Cassettes (ArrayIt) at room temperature (RT) for two hours followed by overnight incubation at 4 °C. The slides were washed in wash buffer (0.05% Tween 20^®^, PBS), and the slides were afterwards incubated with biotinylated detection antibodies (diluted 1:1500, anti-human-CD9, -CD63, -CD81, (Ancell Corporation, MN, USA)) in wash buffer. After a wash, the detection was performed with a subsequent 30 min incubation step with Cy5-labelled streptavidin (diluted 1:1500, Life Technologies) in wash buffer. Prior to scanning at 635 nm, the slides were washed first in washing buffer and then in MilliQ water and dried using a Microarray High-Speed Centrifuge (ArrayIt). The final scanning and spot detection was performed as previously described [34,64].

Data analysis: GraphPad Prism (version 6.04, GraphPad Software, Inc., San Diego, CA, USA) and Excel (version 2013, Microsoft, Redmond, WA, USA) were used to create the graphs. Heat maps were produced using Genesis (version 1.7.6, IGB TU Graz, Graz, Austria). The relative intensities were calculated for a given antibody spot by taking the signal intensity, as the mean signal of triplicate spots, in relation to the sample signal of the negative spot (PBS) in triplicate. For each spot, the signal intensity was calculated by subtracting the mean of the background (no sample/blank, washing buffer) from the mean of the foreground (spot signal). The antibodies signal intensities were converted to log space by log2 transformation before visualization and calculation of linearity.

### 4.6. Statistical Analysis

The statistical analysis of data was performed using SigmaPlot (version 11, Systat Software Inc., San Jose, CA, USA). To test for differences between two groups, a paired or unpaired *t*-test was applied to test for differences between the two groups of cells from the same individual or between two individuals, respectively. To test for differences between more than two groups, a one-way repeated measures ANOVA, followed by Tukey’s post-hoc analysis, was applied. Differences between groups were considered statistically significant, when *p* < 0.05.

## 5. Conclusions

In the current study, we demonstrated that the presence of APCs was required for the existence of HLA-DR on human CD3+ CD4+ T cells. A regulatory phenotype could be induced in CD3+ CD4+ HLA-DR+ T cells after an in vitro antigen-specific immune response. This phenotype was produced both with and without physical T cell:APC contact. Hence, both direct interaction and vesicles can give rise to similar cell populations and may indicate how the immune system facilitates long-distance regulation. Finally, this study presents a novel and relevant methodological platform that can be used to study several features of antigen-specific immune responses related to the contact and intercellular communication between the involved immune cells.
